# The construction of a digital dissemination platform for the intangible cultural heritage using convolutional neural network models

**DOI:** 10.1016/j.heliyon.2024.e40986

**Published:** 2024-12-06

**Authors:** Zhurong Liu

**Affiliations:** School of International Tourism and Culture, Guizhou Normal University, Guiyang, 550025, China

**Keywords:** Densely connected convolutional networks, Bottleneck and compression model, Convolutional neural network, Intangible cultural heritage, Digital dissemination platform, Cultural inheritance

## Abstract

In order to promote the digital dissemination and preservation of Chinese intangible cultural heritage, this work constructs a digital platform for its transmission. The platform integrates a range of advanced technologies, including the Densely Connected Convolutional Networks - Bottleneck and Compression model, a notable convolutional neural network, along with natural language processing algorithms, generative adversarial network algorithms, and neural collaborative filtering algorithms. The platform is validated with 224,055 publicly archived valid data records, ensuring its effectiveness and reliability. The results indicate that the heteroscedasticity-robust standard error is 0.15, and the platform achieves accuracies of 0.87 and 0.89 in 5-fold and 10-fold cross-validation, respectively, demonstrating the model's stability and predictive accuracy. Under various operating conditions, the platform's accuracy, recall, and F1-Score all exceed 0.85, with a root mean square error below 0.12, ensuring efficient and reliable recommendation performance. Regarding user experience, page load times remain steady between 6.45 and 10.25 s, with bounce rates and error rates controlled between 40.88 % to 58.33 % and 0.05 %–0.18 %, respectively. A/B testing and heatmap analysis indicate that optimizing page layout significantly improves click-through and conversion rates. Additionally, in the fields of oral literature and traditional medicine, the platform achieves entity recognition accuracies of 90.2 % and 91.3 %, respectively. The platform also demonstrates strong coverage in the knowledge of folklore and traditional crafts, with rates reaching 92.5 % and 89.8 %, respectively, showcasing its advantages across different domains. This work aims to integrate cultural heritage conservation, cultural inheritance, and digital dissemination, fostering the broad transmission and preservation of intangible cultural heritage in the era of big data.

## 1 Introduction

### 1.1 Research background and motivations

As digital technology continues to advance, the preservation and inheritance of cultural heritage have gained increasing attention [1]. However, traditional ways of transmitting intangible cultural heritage are constrained by factors such as time and space, making it challenging to meet contemporary society's demand for cultural information [2,3]. To enhance the transmission of traditional intangible cultural heritage, it is essential to incorporate digital heritage dissemination methods. At present, the protection and inheritance of cultural heritage have become significant global issues. Countries such as the United States, Germany, China, and the United Kingdom are actively exploring and establishing legal frameworks and management systems for digital heritage. Notably, the launch of the “China-Greece Digital Heritage Joint Laboratory,” a collaboration between China and Greece, signifies the shift of digital heritage protection towards a broader stage of international cooperation. In this context, the interweaving of cultural affinities within cyberspace has made the digital expression of intangible cultural heritage possible.

However, the application of advanced technologies in the preservation and dissemination of intangible cultural heritage faces numerous complex challenges. The inherent diversity and intricate cultural elements of such heritage make the digitalization process particularly demanding. Intangible cultural heritage often embodies deep regional and historical traditions, and a single technological approach is insufficient to fully capture its rich cultural essence. Additionally, for intangible cultural heritage that relies on oral traditions and performances, accurately capturing and reproducing its dynamic characteristics is a critical challenge [[4], [5], [6]]. Moreover, inconsistencies in technological standards, limitations in legal frameworks, and the complexities of international cooperation further complicate the digital protection of cross-border cultural heritage. Throughout this process, careful consideration must be given to privacy protection and cultural sensitivity to ensure that technology effectively balances preservation and protection. For instance, Convolutional Neural Network (CNN)-based image processing methods demonstrate strong recognition capabilities in extracting features from images of intangible cultural heritages. However, when it comes to dynamic cultural performances like traditional dance or theater, these methods struggle to fully capture the fluidity and diversity inherent in such activities. Additionally, language processing technologies excel in text analysis and extracting cultural background information, aiding in the broader dissemination of intangible cultural heritage through written forms. However, they face challenges in accurately interpreting non-standardized languages, dialects, and oral histories. These technologies often fall short when dealing with deep cultural differences, failing to faithfully reproduce the unique essence and subtleties of cultural expression. Although the Generative Adversarial Network (GAN) can simulate and generate realistic cultural images or scenes, they often lack the necessary cultural detail and authenticity when dealing with the complexity of intangible cultural heritage. The digital preservation and dissemination of intangible cultural heritage not only require technological precision but also need to account for cultural diversity and uniqueness, ensuring that digital representations are neither overly simplified nor superficial. Based on this, this work applies CNN models to the construction of a digital dissemination platform for intangible cultural heritage. It integrates Natural Language Processing (NLP) algorithms, GAN, and Neural Collaborative Filtering (NCF) to facilitate both the domestic dissemination and international promotion of intangible cultural heritage based on China's intangible cultural heritage knowledge base. This approach expands the channels and methods of cultural transmission, effectively increasing public awareness and engagement with intangible cultural heritage.

### 1.2 Research objectives

This work aims to develop a digital dissemination platform for intangible cultural heritage using CNN models. The research objectives are as follows: 1) leveraging the Densely Connected Convolutional Networks - Bottleneck and Compression (DenseNet-BC) model for feature extraction of cultural heritage images [7]; 2) employing NLP algorithms to construct a comprehensive knowledge graph; 3) generating realistic cultural heritage images with GAN algorithms; and 4) implementing personalized recommendations through NCF algorithms. The ultimate goal is to create a multifunctional digital dissemination platform that enhances the user experience.

**Fig. 1:**
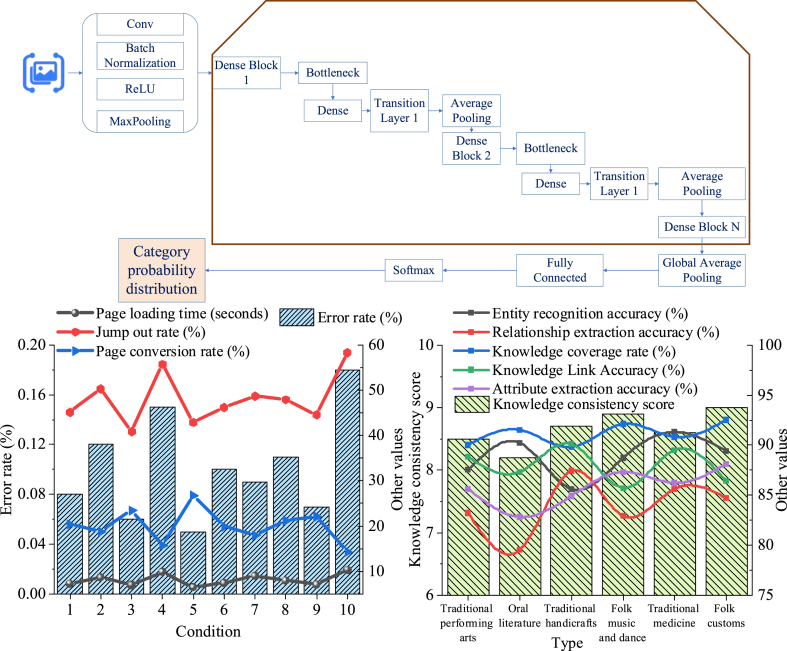
Graphical abstract of this work.

Fig. 1 is a graphical abstract of this work.

## 2 Literature review

Deep learning, a machine learning technique based on artificial neural networks, mimics the way the human brain works to process large volumes of complex data. It has achieved remarkable success in areas such as image recognition and natural language processing. Badawy et al. (2021) explored the importance of promoting educational development through e-learning platforms, particularly how advanced algorithms could extract the topics of learning resources and generate interactive knowledge graphs. They proposed a new algorithm that combined Wikipedia Miner, TextRank, and Gensim, achieving efficient topic extraction and resource linking across different text sizes. The algorithm significantly improved Gensim's accuracy, advancing the process of self-learning and community sustainability [8]. Han and Ghadimi (2022) introduced a hybrid approach based on CNN and Extreme Learning Machines, optimizing the proton exchange membrane fuel cell model using an improved Honey Badger algorithm. The experimental results showed a maximum error rate of 0.039, closely matching the experimental data and outperforming traditional CNN models, demonstrating the potential of artificial intelligence in complex system modeling [9]. Additionally, Abbasihafshejani et al. (2024) investigated methods for detecting selfish behavior in the Algorand blockchain. They used game analysis and mechanism design to successfully increase system throughput, showcasing the potential of artificial intelligence in optimizing distributed systems [10]. In the application of deep learning in digital platform construction, El-Koshiry et al. (2024) examined the effectiveness of deep learning in detecting cyberbullying, particularly the combination of pre-trained word embeddings and focal loss algorithms. The findings reveal that the focal loss algorithm achieved a maximum accuracy of 99 %, highlighting the advantages of deep learning in handling complex text data [11].

The construction of digital platforms relies on the classification and visual presentation of text data. Beyond technological innovation, researchers have conducted more detailed studies on text classification. Khairy et al. (2021) explored the challenges of network language detection in online social networks due to their complexity and resource scarcity. They reviewed 27 studies on Arabic automatic detection systems and analyzed the effectiveness of existing methods. The results revealed that despite the availability of automated solutions for many languages, research on Arabic remained relatively underdeveloped. The shortcomings of existing research emphasize the need for further algorithm optimization and dataset expansion [12]. Mamdouh and Abd-EL-Hafeez (2023) discussed the critical role of text classification in pattern recognition, particularly in classification tasks. Their findings proposed a text classification feature selection technique based on frequent items and relevant items, using association analysis to reduce redundant information and enhance the effectiveness of model construction. The experimental results showed that this technique achieved a high accuracy rate of 95.155 % on a spam SMS dataset, using only 6 % of the features [13]. It demonstrates how innovative feature selection techniques can effectively improve model performance in pattern recognition tasks. In text classification tasks, innovative feature selection techniques can significantly enhance model accuracy and efficiency. The classification and visual presentation of text data are core elements, and therefore, precise and efficient feature selection techniques directly impact platform performance and user experience, providing foundational support for intelligent and personalized platform functions. The aforementioned studies offer valuable technical references for digital platforms in processing and displaying large-scale text data.

In related research on the construction of digital dissemination platforms, Wang et al. (2024) explored the application of multimodal perception systems in contemporary art education. The DenseNet-BC model, based on CNN principles, achieved a 96.15 % accuracy rate in visual feature extraction and task recognition. Their findings advanced digital cultural dissemination and improved the quality and efficiency of art education through an innovative teaching framework and multisensory interactive tasks, thereby optimizing art education [14]. Nagam et al. (2023) found that using DenseNet-BC as an integrated CNN architecture model for strong gravitational lens identification achieved a lower false positive rate compared to ResNets. This provided a more reliable automatic tracking and analysis tool for identification and classification [15]. Arora (2021) developed a method using a DenseNet-based CNN classifier to automatically identify chromosome orientations in chromosome microimages. This innovative method improved the accuracy and efficiency of selecting images for discovering genetic defects and producing karyotypes. Trained on 156,750 microimages, the classifier achieved an error rate of only 1.46 %, indicating its high potential in the field of chromosome imaging [16]. These studies demonstrate the exceptional performance of the DenseNet-BC model across various fields, including art education, astronomy, and cytogenetics. Its high accuracy, low false positive rate, and outstanding application potential offer valuable reference points for constructing a digital dissemination platform for intangible cultural heritage. The model can be employed for visual feature extraction, task recognition, and image classification, thereby enhancing the platform's efficiency, accuracy, and user experience.

Current research has made significant progress in the digital dissemination of intangible cultural heritage, yet it still faces numerous limitations. The DenseNet-BC model excels in visual feature extraction and task recognition but has high computational complexity, making efficient deployment challenging in large-scale data processing and real-time application scenarios. Although GAN algorithms have advantages in generating images, they are constrained by the diversity and quality of training data. This often results in a lack of sufficient diversity and detail realism in the generated results, and are prone to mode collapse issues. When applying NLP techniques to texts related to intangible cultural heritage, researchers frequently encounter challenges such as semantic ambiguity and limitations in cross-domain transfer learning. It leads to insufficient accuracy in knowledge graph construction and depth in semantic analysis. Moreover, existing platforms often rely on traditional collaborative filtering algorithms, which fail to fully explore users' potential preferences and complex interaction patterns, resulting in inadequate precision and practicality in personalized recommendations. To address these issues, this work proposes a more efficient digital dissemination platform for intangible cultural heritage by integrating DenseNet-BC, GANs, NLP, and NCF algorithms. The platform combines the DenseNet-BC model with NLP, GANs, and NCF algorithms to perform fine feature extraction, intelligent semantic analysis, realistic image generation, and personalized recommendations. In this way, the platform achieves high-quality artifact presentation, intelligent search, and immersive interactive experiences.

## 3 Research model

### 3.1 Intangible cultural heritage

**Fig. 2:**
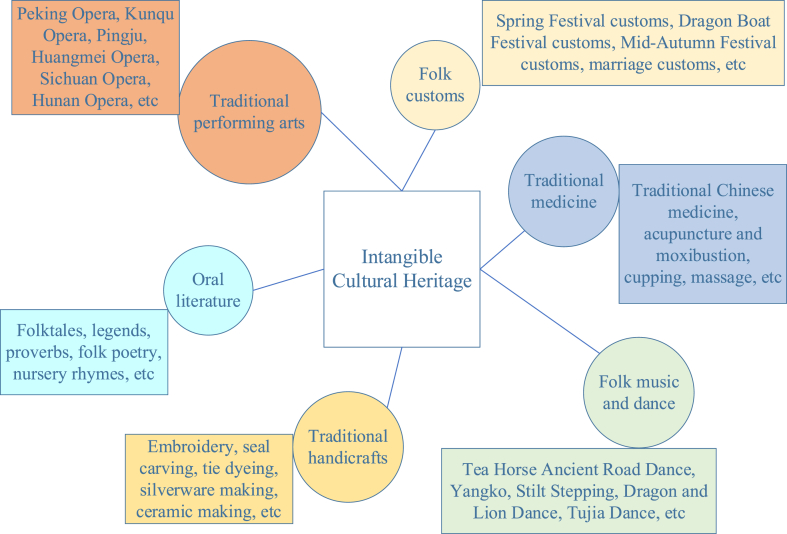
Common types and specific forms of intangible cultural heritage.

Intangible Cultural Heritage refers to cultural traditions created by humans that are passed down through generations and transmitted orally, through practices, and through behaviors. It encompasses various aspects of human social life, including traditional performing arts and oral literature. This heritage represents the cultural characteristics and traditions of different regions, ethnic groups, and historical periods, making it a vital component of human civilization [[17], [18], [19]]. Fig. 2 illustrates common forms of intangible cultural heritage.

### 3.2 Models and algorithms for platform construction under the demand for digital dissemination of intangible cultural heritage

#### 3.2.1 DenseNet-BC model

**Fig. 3:**
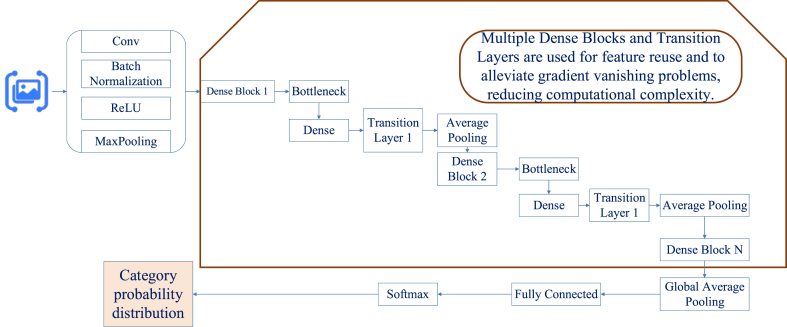
Implementation principle of the DenseNet-BC model.

The DenseNet-BC model, a type of CNN model, is a deep neural network structure built on convolution operations. It achieves feature extraction and representation learning for input data by stacking multiple convolutional and pooling layers [[20], [21], [22]]. The core of DenseNet-BC lies in its “dense connectivity” structure, where each layer receives feature maps from all preceding layers as input and outputs its own feature maps to all subsequent layers. This design significantly enhances feature propagation, reduces information loss within the network, and improves efficiency while mitigating the risk of overfitting. DenseNet-BC also incorporates “bottleneck layers,” which use 1 × 1 convolution operations to reduce the number of feature maps, thereby decreasing computational load and optimizing network performance. Additionally, the model employs compression techniques to reduce the number of feature maps in transition layers, further optimizing storage resources and accelerating data processing. By introducing mechanisms such as dense connections and bottleneck layers, DenseNet-BC improves and optimizes the traditional CNN structure. This advancement boosts parameter efficiency, improves feature transmission, and elevates overall performance [[23], [24], [25]]. Fig. 3 illustrates the implementation principle of the DenseNet-BC model:

Core computational processes of the DenseNet-BC model are as follows:

1) Feature Transmission through Dense Connections

(1) $$X_{l}=H_{l}\left(\left[X_{0},X_{1},\ldots ,X_{l-1}\right]\right)$$

$X_{l}$ represents the output feature map of the l-th layer, $H_{l}$ denotes the dense connection function of the l-th layer, and $\left[X_{0},X_{1},\ldots ,X_{l-1}\right]$ represents the output feature maps of all preceding layers before the l-th layer [26].

2) Feature Extraction through Bottleneck Layers

(2) $$Y_{l}=BN\left(ReLU\left(Conv_{1\times 1}(BN(ReLU(Conv_{3\times 3}\left(X_{l}\right)\right)\right)))))$$

$Y_{l}$ represents the feature map processed by the bottleneck layer, BN denotes the batch normalization operation, ReLU indicates the activation function, and $Conv_{3\times 3}$ represents the 3 × 3 convolution operation.

3) Weight Update in Dense Connections

(3) $$W_{l}^{\left(i,j\right)}=W_{l-1}^{\left(i,j\right)}+\alpha \cdot \frac{\partial L}{\partial W_{l}^{\left(i,j\right)}}$$

$W_{l}^{\left(i,j\right)}$ represents the weight between feature map connections, and $\partial W_{l}^{\left(i,j\right)}$ denotes the gradient of the loss function with respect to the weight.

#### 3.2.2 NLP algorithm

The NLP algorithm is a branch of artificial intelligence designed to understand and process human language [27]. In this context, it is tailored specifically for text data related to intangible cultural heritage, resulting in substantial advancements in semantic analysis [28,29]. By leveraging pre-trained word vector models and transfer learning techniques, the algorithms more effectively leverage language patterns and features within extensive intangible cultural heritage datasets, thereby accelerating the platform's convergence speed [[30], [31], [32]].

The core optimization computations for the NLP algorithms are represented as follows:

(1) Loss function for semantic analysis

(4) $$L_{Y}=-\frac{1}{N}\sum_{i=1}^{N}\;\left[y_{it}\;\log\;\left(p_{it}\right)+\left(1-y_{it}\right)\log\;\left(1-p_{it}\right)\right]$$

$L_{Y}$ represents the loss function for the semantic analysis task; N denotes the number of training samples, $y_{it}$ represents the true label of sample it, and $p_{it}$ denotes the predicted probability of sample it [33].

(2) Transfer learning of word vector models

(5) $$V_{Q}=\alpha V_{Y}+\left(1-\alpha \right)V_{N}$$

$V_{Q}$ represents the transferred word vectors, $V_{Y}$ denotes the pre-trained word vectors, $V_{N}$ signifies the newly trained word vectors, and α denotes the weight parameter for transfer learning [34].

#### 3.2.3 GAN algorithm

**Fig. 4:**
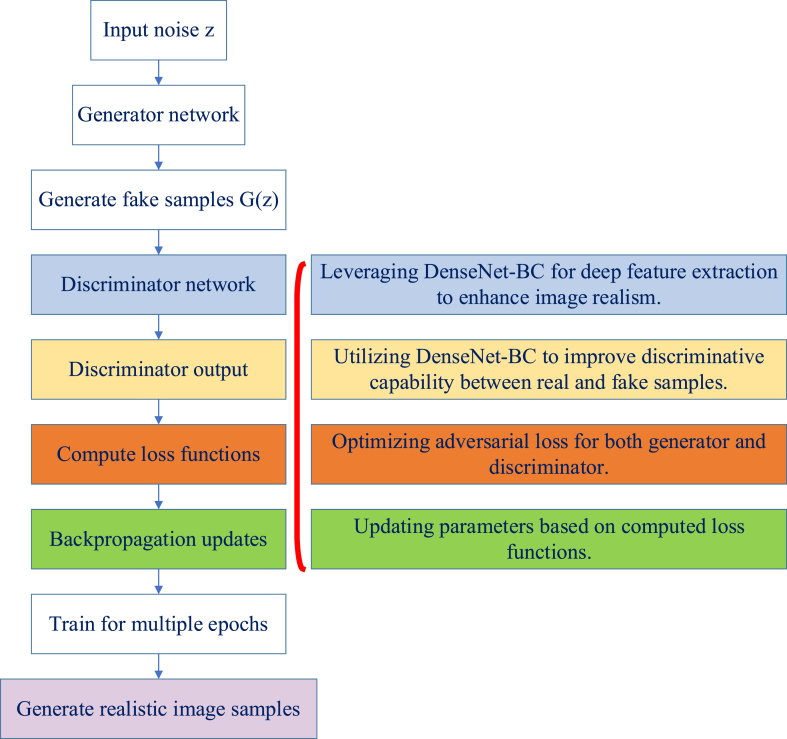
Implementation flowchart of optimized GAN algorithm.

The GAN algorithm is a deep learning framework consisting of a generator and a discriminator. It is designed to produce realistic data samples through adversarial training, where the generator creates samples and the discriminator evaluates their authenticity, driving the generator to improve over time [35]. The GAN algorithm mentioned here is optimized by integrating the DenseNet-BC model into the generator network. The DenseNet-BC model, characterized by its dense connectivity structure and deep feature extraction capabilities, enhances the realism and detail of generated images. This integration allows the generator to produce more realistic and detailed images [36]. Within the GAN framework, DenseNet-BC not only improves the authenticity of the generated images but also increases the efficiency of the discriminator in distinguishing between real and fake images. This combination optimizes the performance of the GAN in both image generation and image recognition, particularly when dealing with complex and high-resolution image data [[37], [38], [39]]. Fig. 4 illustrates the implementation flow of the optimized GAN algorithm.

Core optimization computations of the GAN algorithm include:

(1) Loss function of the generator network

(6) $$L_{S}=-\frac{1}{m}\sum_{i=1}^{m}\;\log\;D\left(G\left(z^{\left(i\right)}\right)\right)$$

$L_{S}$ represents the loss function of the generator network, m denotes the number of training samples, and $z^{\left(i\right)}$ indicates the random noise input vector [40].

(2) The loss function of the discriminator network

(7) $$L_{P}=-\frac{1}{m}\sum_{i=1}^{m}\;\left[\log\;D\left(x^{\left(i\right)}\right)+\log\;\left(1-D\left(G\left(z^{\left(i\right)}\right)\right)\right)\right]$$

$L_{P}$ denotes the loss function of the discriminator network, $x^{\left(i\right)}$ represents real sample images, $D\left(x^{\left(i\right)}\right)$ represents the discriminator's output for real samples, and $G\left(z^{\left(i\right)}\right)$ represents the generator's output for random noise.

(3) Parameter update of the generator network

(8) $$\theta_{G}=\theta_{G}-\alpha \frac{1}{m}\sum_{i=1}^{m}\;\nabla_{\theta_{G}}\;\log\;D\left(G\left(z^{\left(i\right)}\right)\right)$$

$\theta_{G}$ represents the parameters of the generator network, and $\nabla_{\theta_{G}}\;\log\;D(G\left(z^{\left(i\right)}\right)$ denotes the gradient calculation with respect to the generator parameters.

#### 3.2.4 NCF algorithm

This work combines the NCF algorithm with neural networks and collaborative filtering and utilizes the DenseNet-BC model for image feature extraction and NLP for analyzing textual information. This approach constructs implicit representations of users and items [41,42]. NCF goes beyond merely utilizing historical user interaction data by leveraging deep neural networks to learn the latent feature representations of users and items, thereby predicting user preferences more accurately. The algorithm incorporates a Multi-Layer Perceptron (MLP) to learn the user-item interaction function. This approach can capture nonlinear and complex patterns more effectively than traditional matrix factorization or other machine learning methods. Additionally, NCF enhances the model's learning process through end-to-end training and sophisticated loss function design, which improves recommendation accuracy and personalization. This deep feature learning and efficient training strategy enable NCF to deliver more precise and personalized recommendations, significantly boosting user satisfaction and overall system performance [[43], [44], [45]].

Its core optimization calculation is as follows:

(1) Latent representation of users and items

(9) $$h_{u}=\sigma \left(W_{u}\cdot \left[v_{u};e_{u}\right]\right)$$

(10) $$h_{i}=\sigma \left(W_{i}\cdot \left[v_{i};e_{i}\right]\right)$$

$h_{u}$ and $h_{i}$ respectively denote the latent representation vectors for user u and item i; $v_{u}$ and $v_{i}$ represent the feature vectors for u and i; $e_{u}$ and $e_{i}$ denote the embedding vectors for u and i, and $W_{u}$ and $W_{i}$ represent the corresponding weight matrices [46,47].

(2) User-item interest prediction

(11) $${\hat{y}}_{u,i}=h_{u}^{T}\cdot h_{i}$$

${\hat{y}}_{u,i}$ represents the predicted interest of user u for item i, and $h_{u}^{T}$ denotes the transpose of the user's latent representation vector.

(3) Loss Function

(12) $$L=-\sum_{\left(u,i\right)\in D}\;y_{u,i}\;\log\;\left({\hat{y}}_{u,i}\right)+\left(1-y_{u,i}\right)\log\;\left(1-{\hat{y}}_{u,i}\right)$$

D denotes the training set, and $y_{u,i}$ represents the true interest label of user u for item i.

## 4 Experimental design and performance evaluation

When constructing a digital dissemination platform for Chinese intangible cultural heritage, integrating the DenseNet-BC model with NLP, GAN, and NCF algorithms enables a versatile approach. This integration enhances human-computer interaction, supports personalized content delivery, and creates immersive experiences.

The platform's foundation is the DenseNet-BC model, which utilizes its unique dense connectivity structure for precise feature extraction from images of intangible cultural heritage artifacts. The strength of DenseNet-BC lies in its ability to effectively utilize all features from previous layers, reducing the number of parameters through feature reuse and enhancing computational efficiency. This can enhance the model's ability to recognize image content while preserving intricate details. By deeply extracting features from cultural relic images, the platform can obtain rich visual information, providing a solid foundation for subsequent image processing and data analysis [48,49]. Building on this, the GAN algorithm generates high-fidelity images and performs color rendering through adversarial networks, restoring the historical appearance of artifacts and delivering realistic visual effects. The generator strives to create images as close to reality as possible, while the discriminator works to distinguish between real and generated images, with this adversarial process driving continuous system optimization. Meanwhile, the NLP algorithm handles and interprets text data related to intangible cultural heritage, constructing a rich knowledge graph that links the history, context, and cultural significance of artifacts. Through semantic analysis, the NLP algorithm enables intelligent search and content recommendation, providing users with precise cultural background information. Finally, the collaborative filtering algorithm personalizes recommendations based on user behavior data. Leveraging the NCF algorithm, it analyzes user behavior and preferences to deliver tailored content recommendations [50]. By utilizing past interaction data, the algorithm predicts which aspects of intangible cultural heritage may interest the user, thereby offering a personalized recommendation list.

Table 1 presents the core pseudocode for this process.

**Table 1:**
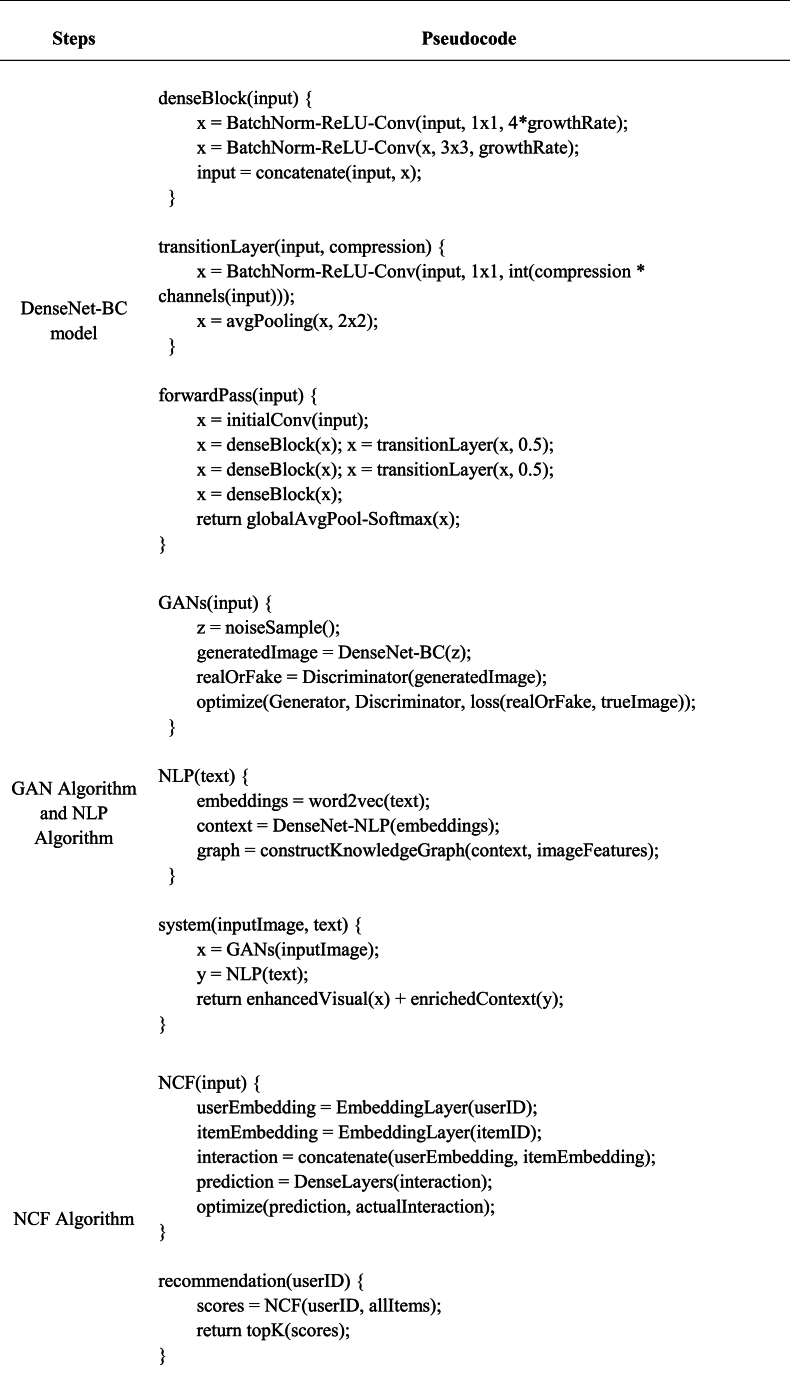
Pseudocode for Implementing the Digital Dissemination Platform for Chinese Intangible Cultural Heritage.

By integrating multiple algorithms, this digital dissemination platform for intangible cultural heritage not only enables high-quality artifact displays but also offers intelligent search, personalized recommendations, and immersive interactive experiences. This comprehensive approach significantly enhances user engagement and satisfaction. The platform seamlessly combines artifact preservation, cultural heritage transmission, and digital dissemination, driving the widespread promotion and protection of intangible cultural heritage in the era of big data. By integrating image features extracted using DenseNet-BC with textual analysis performed by NLP, the NCF algorithm recommends intangible cultural heritage content tailored to users' interests. This approach not only aligns recommendations with user preferences but also boosts engagement and satisfaction.

### 4.1 Datasets collection

This work utilizes text and image information from public datasets, selecting them according to the digital representation needs of intangible cultural heritage to ensure high quality, representativeness, and comprehensiveness. The selection criteria include ensuring that the data covers rich cultural content, including key dimensions such as historical background, technical details, and cultural value, ensuring both depth and breadth in cultural expression. Priority is given to data from authoritative, nationally or internationally recognized cultural heritage databases to ensure reliability. Additionally, diversity of data is also crucial, encompassing multimedia formats such as text, images, audio, and video to provide a comprehensive representation of intangible cultural heritage from multiple perspectives. To ensure data authenticity and completeness, special attention is given to avoiding records with significant gaps or ambiguous information. Additionally, data processability is a key consideration. Priority is given to data that has already been preliminarily organized and is suitable for further analysis and modeling, ensuring a solid foundation for building the digital dissemination platform.

Initially, a total of 372,037 data entries are obtained. The work pre-processes the data to remove unusable entries. Specifically, text data are processed using tokenization and stop-word removal techniques to break long texts into meaningful words, followed by semantic analysis to extract key themes and concepts. For image data, image size normalization is applied, adjusting all images to a consistent dimension of 224 × 224 pixels to ensure uniform input. Following this, histogram equalization is used to enhance image contrast and improve color representation. Audio data are standardized to a 44.1 kHz sampling rate and key audio features are extracted using Mel-Frequency Cepstral Coefficients (MFCC). Video data are compressed using H.264 encoding while maintaining a resolution of 720p for clarity. After processing, 224,055 valid data entries remain. Table 2 provides information on the dataset.

**Table 2:** Research dataset.

Type	Dataset Features	Data Volume	Dataset Link	Training/Testing
Traditional Performing Arts	Recordings and videos of various performing arts, including drama and storytelling.	13000	https://www.china-nonheritage.cn/	11000/2000
	Text and multimedia materials covering traditional performing arts from different regions of China.	14000	http://www.chinaculture.org/gb/cn/	11200/2800
Oral Literature	Collection of Chinese folk oral literature works and related research.	15000	https://www.cnki.net/	12000/3000
	Academic papers and reviews related to oral literature.	18005	http://www.cqvip.com/	14500/3505
Traditional Handicrafts	Demonstrations of traditional handicraft techniques and works.	15050	https://www.intangibleheritage.org/	12500/2550
	Cases of traditional craft inheritance and innovative applications.	25000	http://www.ihchina.cn/	17500/7500
Folk Music and Dance	Audio and video materials of folk music and dance, along with background stories.	17000	https://www.nlc.cn/	13600/3400
	Multimedia resources and research data on Chinese folk music and dance.	22000	https://www.cfsdc.org/	17600/4400
Traditional Medicine	Classic texts and image data related to traditional medicine.	22000	http://www.mctcm.org/	17600/4400
	Research literature and application cases on Traditional Chinese Medicine.	36000	https://www.chinamedicalnews.com/	28800/7200
Folk Customs	Cultural connotations of local folk activities and festivals.	15000	https://www.mct.gov.cn/	12000/3000
	Historical data and customs from various regions of China.	12000	http://www.ncha.gov.cn/	9600/2400

### 4.2 Experimental environment

Table 3 displays the experimental environment.

**Table 3:** Experimental environment.

Type	Environment	Parameters
Hardware	CPU	Intel Core i7-10700K
	GPU	NVIDIA GeForce RTX 3080
	Memory	32 GB DDR4
	Storage	1 TB SSD
Software	Operating System	Windows 10
	Programming Language	Python 3.8
	Deep Learning Frameworks	TensorFlow 2.5
		PyTorch 1.9
		Keras 2.4

### 4.3 Parameters setting

First, the platform undergoes robustness testing using heteroskedasticity-robust standard errors, cluster-robust standard errors, and k-fold cross-validation. Table 4 outlines the parameters for the robustness testing environment.

**Table 4:** Robustness testing environment parameters.

Parameter	Value
Dataset	Intangible Cultural Heritage Dataset
Model	Deep Learning Model
Feature Extraction Method	DenseNet-BC
Training Epochs	100
Batch Size	64
Learning Rate	0.001
Loss Function	Mean Squared Error
Optimizer	Adam

After completing 100 rounds of deep learning model training, heteroscedasticity-robust standard errors, clustering-robust standard errors, and k-fold cross-validation, the robustness testing analysis takes approximately 51 h in total. Each training round lasts about 30 min, and the overall process includes data processing and robustness calculations. With the platform's stability established, the focus shifts to analyzing the recommendation system's performance, user experience, and the accuracy of the knowledge graph. To assess the recommendation system, performance is evaluated under six distinct conditions, each with notable differences. Table 5 provides the specific parameters for these conditions. Table 5 details the specific parameters for these six conditions.

**Table 5:** Parameters for the analysis of 6 conditions in the recommendation system performance.

Condition	Parameter settings of DenseNet-BC Model	Parameter settings of GAN algorithms	Parameter settings of NLP algorithms
Condition A	Network Depth: 121 layers	The Number of Generator and Discriminator Layers: 4 layers	Text Vectorization Method: Word2Vec
	Growth Rate: 32	Loss Function: Wasserstein GAN (WGAN)	Embedding Dimension: 300
	Compression Factor: 0.5	Learning Rate: 0.0002	Maximum Text Length: 1000 words
	Image Input Size: 224 × 224 pixels	Batch Size: 64	Training Epochs: 50
Condition B	Network Depth: 169 layers	The Number of Generator and Discriminator Layers: 6 layers	Text Vectorization Method: GloVe
	Growth Rate: 32	Loss Function: Least Squares GAN (LSGAN)	Embedding Dimension: 200
	Compression Factor: 0.5	Learning Rate: 0.0001	Maximum Text Length: 800 words
	Image Input Size: 256 × 256 pixels	Batch Size: 128	Training Epochs: 100
Condition C	Network Depth: 201 layers	The Number of Generator and Discriminator Layers: 8 layers	Text Vectorization Method: FastText
	Growth Rate: 32	Loss Function: BCEWithLogitsLoss	Embedding Dimension: 100
	Compression Factor: 0.5	Learning Rate: 0.0003	Maximum Text Length: 1200 words
	Image Input Size: 192 × 192 pixels	Batch Size: 256	Training Epochs: 80
Condition D	Network Depth: 161 layers	The Number of Generator and Discriminator Layers: 10 layers	Text Vectorization Method: BERT
	Growth Rate: 32	Loss Function: Hinge Loss	Embedding Dimension: 512
	Compression Factor: 0.5	Learning Rate: 0.0004	Maximum Text Length: 1500 words
	Image Input Size: 320 × 320 pixels	Batch Size: 512	Training Epochs: 120
Condition E	Network Depth: 121 layers	The Number of Generator and Discriminator Layers: 4 layers	Text Vectorization Method: Word2Vec
	Growth Rate: 32	Loss Function: Wasserstein GAN (WGAN)	Embedding Dimension: 300
	Compression Factor: 0.5	Learning Rate: 0.0002	Maximum Text Length: 1000 words
	Image Input Size: 224 × 224 pixels	Batch Size: 64	Training Epochs: 50
Condition F	Network Depth: 169 layers	The Number of Generator and Discriminator Layers: 6 layers	Text Vectorization Method: GloVe
	Growth Rate: 32	Loss Function: Least Squares GAN (LSGAN)	Embedding Dimension: 200
	Compression Factor: 0.5	Learning Rate: 0.0001	Maximum Text Length: 800 words
	Image Input Size: 256 × 256 pixels	Batch Size: 128	Training Epochs: 100

Under six different conditions, the DenseNet-BC model, along with the GANs and NLP algorithms, is configured and trained separately. The total time spent on training and analysis for each condition is approximately 6.5 h, with an average training time of 60 min per condition. User experience analysis aims to verify platform performance under non-optimal conditions and test user sensory experience in potentially adverse environments, while adapting to common environmental conditions. Table 6 lists the specific parameters for 10 conditions.

**Table 6:** Parameters for user experience analysis under 10 conditions.

Condition	User Traffic	Network Speed (Mbps)	Device Type	Device Resolution (pixels)	Network Stability	Network Latency (ms)	Platform Loading Time (s)
1	10000	5	Mobile	720 × 1280	Low	200	100
2	8000	10	Tablet	1024 × 768	Low	150	80
3	12000	3	PC	1366 × 768	Low	250	120
4	5000	1	Mobile	480 × 800	Low	300	150
5	15000	8	Tablet	1280 × 800	Low	180	90
6	10000	2	PC	1280 × 1024	Low	280	130
7	7000	6	Mobile	540 × 960	Low	220	110
8	9000	4	Tablet	800 × 1280	Low	200	100
9	11000	2	PC	1024 × 768	Low	270	140
10	4000	0.5	Mobile	320 × 480	Low	350	180

The user experience tests, which cover loading times, bounce rates, and page conversion rates under ten different network and device conditions, take a total of approximately 11 h. Following this, an accuracy analysis of the platform's knowledge graph is conducted. Given the complexity of entity recognition, relationship extraction, and attribute extraction for the construction of knowledge graphs related to different types of intangible cultural heritage, the accuracy analysis of the knowledge graph requires about 18 h. To provide a clearer representation of the knowledge graph's accuracy, the work introduces a parameter called the knowledge consistency score. The knowledge consistency score evaluates the completeness and accuracy of knowledge by comparing the consistency of relationships and attributes among entities in the knowledge graph, with a score range from 1 to 10. The calculation reads:

(13) $$S=\frac{\sum_{i=1}^{nt}\;\left(\frac{1}{r_{i}}\cdot \frac{1}{p_{i}}\cdot \frac{1}{a_{i}}\right)}{nt}$$

nt represents the number of entities in the knowledge graph, $r_{i}$ denotes the relationship extraction accuracy of the i-th entity, $p_{i}$ represents the knowledge coverage rate of the i-th entity, and $a_{i}$ indicates the attribute extraction accuracy of the i-th entity. This equation considers the comprehensive factors of inter-entity relationships, knowledge coverage, and attribute extraction accuracy to evaluate the consistency and quality of the knowledge graph.

### 4.4 Performance evaluation

#### 4.4.1 Platform robustness test analysis

**Fig. 5:**
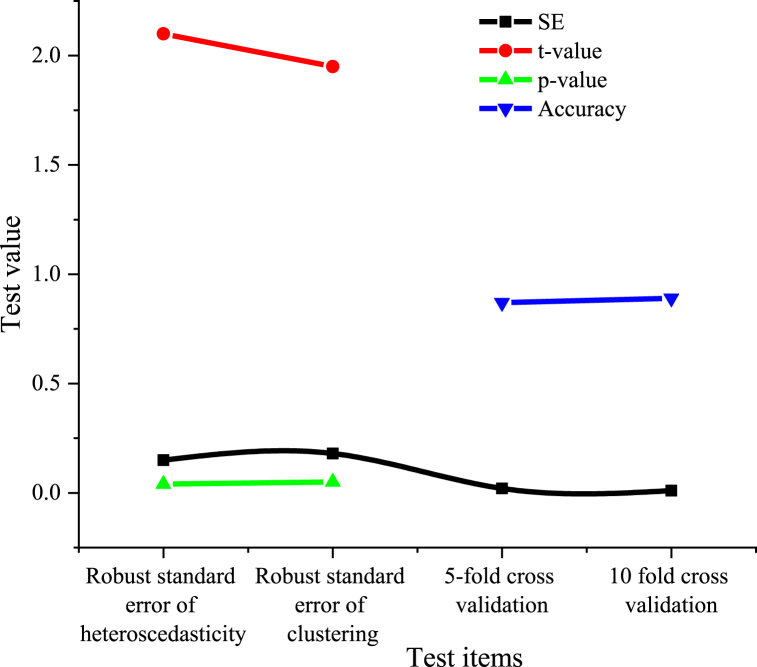
The robustness analysis of the digital dissemination platform for intangible cultural heritage.

Fig. 5 shows the results of the platform's robustness test analysis.

Fig. 5 illustrates that the heteroscedasticity-robust standard error is 0.15, accompanied by a t-value of 2.10 and a p-value of 0.04. The cluster-robust standard error is 0.18, with a t-value of 1.95 and a p-value of 0.05. The 5-fold cross-validation accuracy stands at 0.87, with a standard error of 0.02, while the 10-fold cross-validation accuracy is 0.89, with a standard error of 0.01. Analyzing the range of standard errors along with the statistical measures of t-values and p-values indicates that the platform demonstrates significant robustness in both heteroscedasticity and cluster robustness, with statistical significance (p < 0.05). This underscores a high degree of confidence in the results. Additionally, the cross-validation accuracy analysis reveals that the platform performs effectively in prediction accuracy, supported by low standard errors, thereby demonstrating a high level of result reliability.

#### 4.4.2 Recommendation system performance analysis

**Fig. 6:**
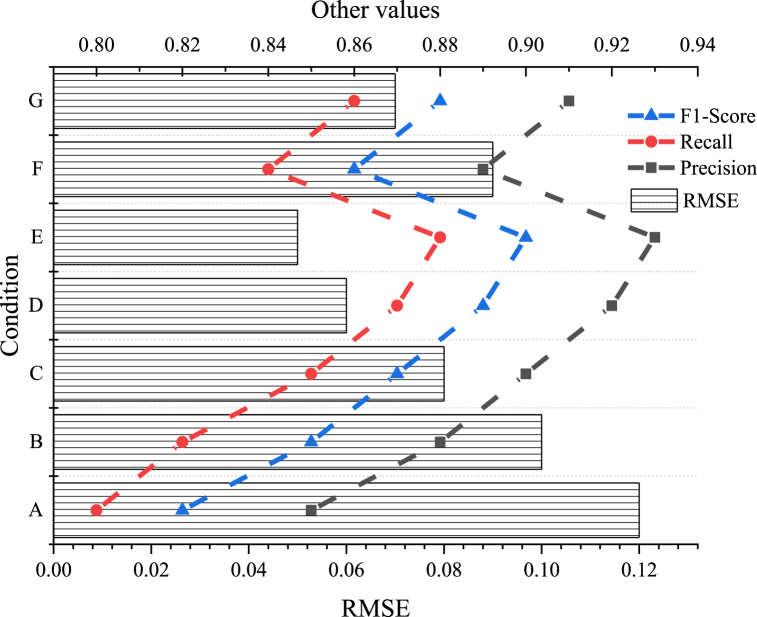
Recommendation system performance analysis of the digital dissemination platform for intangible cultural heritage.

Fig. 6 presents the results of the recommendation system performance analysis.

Fig. 6 illustrates how the system's performance varies with different conditions. Under condition 5, the system demonstrates optimal performance with a precision of 0.93, recall of 0.88, and F1-Score of 0.90. Meanwhile, the root mean square error (RMSE) is only 0.05, indicating that under this condition, the system offers highly accurate and personalized recommendations that closely match users' needs with minimal error. Although the system's performance varies under other conditions, it generally maintains good stability and reliability, with precision, recall, and F1-Score consistently above 0.85 and RMSE below 0.12. This suggests that the system can provide competitive recommendation performance across diverse conditions. Overall, the platform's recommendation system demonstrates superior performance under various conditions, delivering high-quality personalized recommendations that enhance user satisfaction and engagement.

#### 4.4.3 User experience analysis

**Fig. 7:**
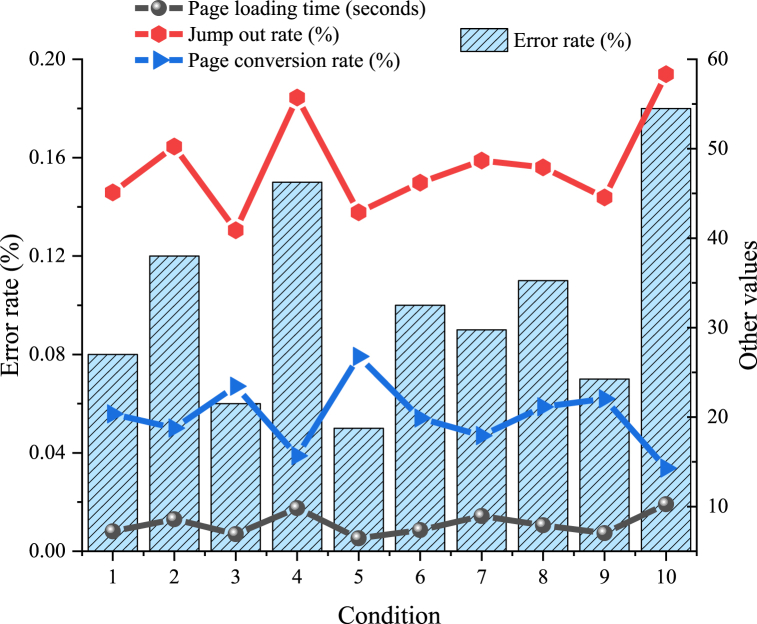
Analysis of user experience on the digital dissemination platform for intangible cultural heritage.

Fig. 7 presents the results of the user experience analysis.

Fig. 7 reveals that as the number of user entries increases, the page loading time fluctuates but overall stabilizes, ranging between 6.45 s and 10.25 s. However, the bounce rate and error rate show an increasing fluctuation trend, with the bounce rate ranging from 40.88 % to 58.33 % and the error rate between 0.05 % and 0.18 %. Meanwhile, the page conversion rate exhibits a fluctuating growth trend, ranging from 15.67 % to 26.78 %. A/B testing and heatmap analysis further reveal that page layout and design significantly influence user behavior and preferences. For instance, click rates are higher in the top and bottom areas of the page compared to the middle sections.

From the comprehensive analysis of the data above, the following conclusions can be drawn: despite some differences in user experience under different conditions, the platform demonstrates advantageous performance across various metrics. Page loading time remains relatively stable within an acceptable range, while the bounce rate is relatively low and the page conversion rate is relatively high. These findings suggest that the platform effectively attracts and retains users.

Under the same preset conditions, A/B testing and heatmap analysis are conducted separately. Table 7 presents the results.

**Table 7:** A/B test and heatmap analysis results.

User Traffic	A/B Test Result	Heatmap Analysis
10000	B	High click area at the bottom of the page
8000	A	Lower click rate in the middle section
12000	A	High click area at the top of the page
5000	B	Slow page loading speed
15000	A	Overall higher click-through rate in the page
10000	B	Low conversion rate at the top of the page
7000	A	Higher click rate at the bottom of the page
9000	B	Moderate page loading speed
11000	A	Overall higher conversion rate of the page
4000	B	Higher error rate on the page

Table 7 suggests that A/B testing results show a concentration of user clicks in the bottom and top areas of the page, while the middle section experiences a lower click-through rate. This indicates that page layout and design significantly influence user click behavior, suggesting that adjusting the page layout could enhance user engagement. Additionally, heatmap analysis confirms a generally higher click-through rate in the bottom and top sections, indicating user preference for browsing content in these areas. Consequently, optimizing the page layout and content placement, with a focus on the bottom and top sections, could effectively improve both click-through and conversion rates.

#### 4.4.4 Knowledge graph accuracy analysis

**Fig. 8:**
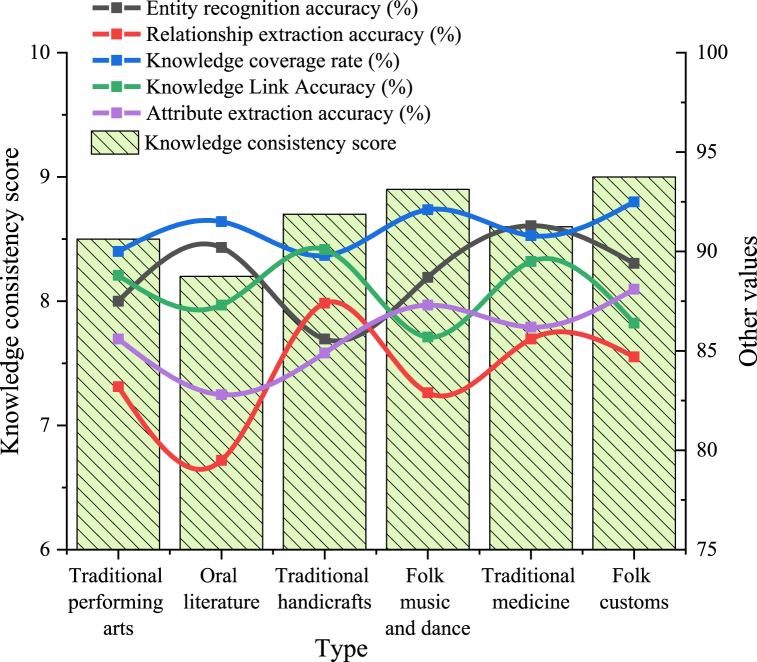
Knowledge graph accuracy analysis of the digital dissemination platform for intangible cultural heritage.

Fig. 8 illustrates the results of the knowledge graph accuracy analysis.

Fig. 8 displays the overall accuracy of the knowledge graph across various types of intangible cultural heritage. The platform consistently delivers strong performance in accuracy. Entity recognition is particularly precise in oral literature and traditional medicine, achieving rates of 90.2 % and 91.3 %, respectively. Moreover, folk customs and traditional crafts exhibit high knowledge coverage rates of 92.5 % and 89.8 %, respectively. Additionally, folk customs achieves the highest score of 9 points in knowledge consistency evaluation. In summary, the platform demonstrates high accuracy and consistency across different domains of intangible cultural heritage, providing reliable cultural knowledge services to users. The platform's strong performance in diverse intangible cultural heritage areas highlights the effectiveness and stability of its knowledge graph construction and management, fulfilling users' needs for accurate and comprehensive cultural information.

### 4.5 Discussion

This work introduces the DenseNet-BC model to construct a digital dissemination platform for intangible cultural heritage. This model provides multi-level and multifunctional digital dissemination capabilities, enhancing user experience and promoting the inheritance and protection of intangible cultural heritage. Through the integrated application of DenseNet-BC, NLP, GAN, and NCF algorithms, adjustments are made to the network architectures and parameter settings of generators and discriminators. Optimization of the loss function design is implemented, and training is conducted using higher resolution image datasets to improve the clarity and detail representation of generated images. This enhancement boosts the visual effects and realism of cultural artifact images. The platform thus supports functionalities such as artifact exhibition, intelligent search, and personalized recommendations, thereby improving the efficiency and quality of intangible cultural heritage dissemination. However, from a critical perspective, despite the significant positive results achieved, some factors may affect the comprehensiveness of the findings during application. For example, users' familiarity with digital tools may vary across different cultural backgrounds, potentially influencing their ability to fully utilize the platform's features. Additionally, cultural differences might subtly impact users' understanding and acceptance, thereby affecting the dissemination effectiveness to some extent. Western users may be more accustomed to interactive and visual interface designs, while some Asian users may place greater emphasis on content depth and historical context. For platform applications in multicultural environments, it is essential to make corresponding adjustments in interface design, content presentation, and feature recommendations to meet the needs of users from diverse cultural backgrounds. Although algorithm optimization has improved the platform's performance, its universality and adaptability in different technological environments still need further consideration. From the user's perspective, their familiarity with digital tools and understanding of technology applications significantly influence the acceptance of platform functionalities and the effectiveness of dissemination. Experienced users can fully leverage features such as personalized recommendations and knowledge graphs, while users who are less familiar with technology may require more guidance and training modules. This necessitates a more inclusive platform design that lowers barriers to use and ensures a balance between technology and user experience. Therefore, future research should explore these potential contextual factors more thoroughly to ensure that the platform maximizes its utility across diverse user groups while continuing to play a central role in the digital dissemination of intangible cultural heritage.

## 5 Conclusion

### 5.1 Research contribution

The analysis of the results shows that the platform exhibits high reliability in both heteroscedasticity and cluster robustness tests. Under condition 5, the recommendation system achieves an accuracy of 0.93 and an F1-Score of 0.90. The page loading time remains stable between 6.45 and 10.25 s, and the entity recognition accuracy of the knowledge graph reaches up to 91.3 %. This work demonstrates the platform's superior performance in robustness, recommendation efficacy, user experience, and knowledge graph accuracy through the construction of a digital dissemination platform for intangible cultural heritage. Referencing previous literature reviews, this work compares the implementation and results of three cutting-edge literature models with the model presented. Table 8 presents the comparison results.

**Table 8:** Comparison of results between the model proposed and other advanced models.

Study	Model	Implementation Principle	Results	Comparison with This Work
Arora (2021) [51]	DenseNet	It is a DenseNet-based classifier to automatically identify orientations in chromosome images, reducing errors and improving identification efficiency.	The error rate of 1.46 %, improving accuracy and efficiency in chromosome imaging.	While both studies use DenseNet, this work focuses on digital dissemination and user experience in cultural heritage rather than biomedical image classification.
Nagam et al. (2023) [52]	DenseNet-BC + Ensemble Model	It applies the DenseNet-BC ensemble model to identify strong gravitational lensing, achieving low false positive rates while maintaining high true positive rates.	Low false positive rate and high true positive rate, offering more reliable tools for automatic tracking and analysis.	Compared to Nagam et al. this work employs DenseNet-BC for intangible cultural heritage image classification and recommendation, emphasizing cultural heritage dissemination and educational applications.
Wang et al. (2024) [53]	DenseNet-BC	Visual feature extraction based on CNN, a multimodal perception system applied to art education	The task recognition accuracy of 96.15 %, enhancing the quality and efficiency of art education.	Although the same DenseNet-BC model is used for visual feature extraction, this work focuses more on the digital dissemination and preservation of intangible cultural heritage
This work	DenseNet-BC + GAN + NLP + NCF	Integration of DenseNet-BC, GAN, NLP, and NCF algorithms to build a digital dissemination platform for intangible cultural heritage	The platform's visual feature extraction, task recognition, image generation, and user personalization recommendation functions are improved, comprehensively enhancing user experience and cultural dissemination efficiency.	This work not only utilizes DenseNet-BC but also integrates GAN, NLP, and NCF algorithms to comprehensively improve the digital dissemination and preservation of intangible cultural heritage.

Table 8 shows that in the construction of the digital dissemination platform for intangible cultural heritage, this work integrates DenseNet-BC, GAN, NLP, and NCF algorithms. Compared to Wang et al. (2024), who used only DenseNet-BC in a multimodal perception system, this work further enhances image generation and user recommendation functions based on visual feature extraction. In contrast to the studies by Nagam et al. (2023) and Arora (2021), which focused on model accuracy and low false positive rates, this work demonstrates broader application value in optimizing the overall performance and user experience of the platform.

Overall, the innovative contributions of this work lie in significantly improving the digital dissemination platform for intangible cultural heritage by introducing CNN. This achieves multi-layered, multi-functional digital dissemination, and deepens understanding and practice in the field of intangible cultural heritage preservation. Specifically, the novelty is reflected in two aspects: (1) Novelty in Main Contribution: This work applies deep learning technology to cultural heritage preservation. The introduction of the CNN model not only enhances the accessibility and dissemination efficiency of cultural content but also improves the quality and interactivity of cultural information through precise feature extraction and data processing techniques; (2) Novelty in Scenario Contribution: The work optimizes the platform's user experience through the integrated application of CNN and other advanced algorithms, providing a personalized and immersive interactive environment, and enhancing the breadth and depth of cultural dissemination. This is particularly notable in comprehensive coverage and longitudinal cultivation of intangible cultural heritage dissemination. Additionally, experimental results confirm the platform's superior performance in operational stability, information accuracy, and user engagement, significantly improving the digital protection and dissemination of intangible cultural heritage. Through these innovative applications, the platform not only serves as a powerful tool for preserving intangible cultural wealth but also offers new perspectives and practical pathways for digital heritage protection. It enhances the understanding and acceptance of advanced technology applications in the field of cultural heritage preservation and greatly promotes the protection and sustainable use of digital cultural assets globally.

### 5.2 Future works and research limitations

The present research work has the following shortcomings: (1) Further exploration is needed for fine-tuning the application of various algorithms in the intangible cultural heritage platform. (2) Evaluation of platform user experience and interactivity remains incomplete. Future improvements could focus on optimizing algorithm parameters, analyzing user feedback data, and enhancing platform functionality and performance, while also improving user experience and interactivity. (3) Currently, the platform research primarily focuses on China's intangible cultural heritage. While maintaining its effectiveness in the existing regions, future efforts should aim to continuously enrich and test the platform's effectiveness in global intangible cultural heritage applications.

To address this, future research will introduce fine-grained user behavior analysis models to delve into user interaction data on the platform. These data include key parameters such as click-through rates, dwell time, and task completion rates, to quantify user experience and interactivity. Additionally, the A/B testing method will be retained to experimentally compare different versions of the interface and functional modules, optimizing the algorithm's response speed and recommendation accuracy based on user feedback data. Furthermore, the platform will integrate sentiment analysis technology to identify users' emotional tendencies during use, dynamically adjusting the interface layout and content presentation. Through testing across different cultural heritages, the platform's applicability and stability in diverse cultural contexts will be verified. This approach aims to expand its effective dissemination and application globally, providing more comprehensive technical support for the digital preservation of intangible cultural heritage while stabilizing the digital expression of domestic intangible cultural heritage.

## Data availability statement

Data will be made available on request.

## Declaration of competing interest

The authors declare that they have no known competing financial interests or personal relationships that could have appeared to influence the work reported in this paper.
